# Predicting Lymph Node Metastasis Status from Primary Muscle-Invasive Bladder Cancer Histology Slides Using Deep Learning: A Retrospective Multicenter Study

**DOI:** 10.3390/cancers15113000

**Published:** 2023-05-31

**Authors:** Qingyuan Zheng, Jun Jian, Jingsong Wang, Kai Wang, Junjie Fan, Huazhen Xu, Xinmiao Ni, Song Yang, Jingping Yuan, Jiejun Wu, Panpan Jiao, Rui Yang, Zhiyuan Chen, Xiuheng Liu, Lei Wang

**Affiliations:** 1 Department of Urology, Renmin Hospital of Wuhan University, Wuhan 430060, China; zqy710890394@whu.edu.cn (Q.Z.);; 2 Institute of Urologic Disease, Renmin Hospital of Wuhan University, Wuhan 430060, China; 3 Department of Urology, People’s Hospital of Hanchuan City, Xiaogan 432300, China; 4 University of Chinese Academy of Sciences, Beijing 100049, China; 5 Trusted Computing and Information Assurance Laboratory, Institute of Software, Chinese Academy of Sciences, Beijing 100190, China; 6 Department of Pharmacology, School of Basic Medical Sciences, Wuhan University, Wuhan 430072, China; 7 Department of Pathology, Renmin Hospital of Wuhan University, Wuhan 430060, China

**Keywords:** muscle-invasive bladder cancer, lymph node metastasis, deep learning, whole slide image, new predictive biomarker

## Abstract

**Simple Summary:**

Accurate prediction of lymph node metastasis (LNM) status in patients with muscle-invasive bladder cancer (MIBC) before radical cystectomy can guide the use of neoadjuvant chemotherapy and the extent of pelvic lymph node dissection. However, there are no reliable tools available for achieving this goal. Data-driven deep-learning techniques have been widely used in disease diagnosis, prognosis assessment, and treatment response prediction by identifying subtle patterns in digitized histopathological images. In this study, we developed a weakly-supervised model based on multiple instance learning and attention mechanism for predicting LNM status in MIBC patients, demonstrating decent performance in three independent cohorts. The visualization technique revealed that the stroma surrounding the tumor with lymphocytic inflammation seemed to be the critical feature for predicting LNM. This deep learning-based study provides a non-invasive and low-cost preoperative prediction tool for identifying MIBC patients with a high risk of LNM.

**Abstract:**

Background: Accurate prediction of lymph node metastasis (LNM) status in patients with muscle-invasive bladder cancer (MIBC) before radical cystectomy can guide the use of neoadjuvant chemotherapy and the extent of pelvic lymph node dissection. We aimed to develop and validate a weakly-supervised deep learning model to predict LNM status from digitized histopathological slides in MIBC. Methods: We trained a multiple instance learning model with an attention mechanism (namely SBLNP) from a cohort of 323 patients in the TCGA cohort. In parallel, we collected corresponding clinical information to construct a logistic regression model. Subsequently, the score predicted by the SBLNP was incorporated into the logistic regression model. In total, 417 WSIs from 139 patients in the RHWU cohort and 230 WSIs from 78 patients in the PHHC cohort were used as independent external validation sets. Results: In the TCGA cohort, the SBLNP achieved an AUROC of 0.811 (95% confidence interval [CI], 0.771–0.855), the clinical classifier achieved an AUROC of 0.697 (95% CI, 0.661–0.728) and the combined classifier yielded an improvement to 0.864 (95% CI, 0.827–0.906). Encouragingly, the SBLNP still maintained high performance in the RHWU cohort and PHHC cohort, with an AUROC of 0.762 (95% CI, 0.725–0.801) and 0.746 (95% CI, 0.687–0.799), respectively. Moreover, the interpretability of SBLNP identified stroma with lymphocytic inflammation as a key feature of predicting LNM presence. Conclusions: Our proposed weakly-supervised deep learning model can predict the LNM status of MIBC patients from routine WSIs, demonstrating decent generalization performance and holding promise for clinical implementation.

## 1. Introduction

Bladder cancer is the most commonly diagnosed urological malignancy worldwide, with approximately 573,278 new cases and 212,536 deaths in 2020 [1]. Based on the depth of invasion, bladder cancer can be classified into non-muscle invasive bladder cancer and muscle-invasive bladder cancer (MIBC) [2]. Generally, MIBC has a poorer prognosis and a higher recurrence rate, requiring more aggressive diagnosis and treatment [3]. Transurethral resection of bladder tumor (TURBT) with histopathological examination is the gold standard for diagnosis and staging in clinical practice [4]. For patients diagnosed with localized MIBC, guidelines recommend radical cystectomy (RC) and pelvic lymph node dissection (PLND) as the standard treatment [5]. Although RC achieves negative surgical margins in the vast majority of patients, the disease recurrence rate remains high at approximately 50%, suggesting the presence of extravesical tumor deposits at the time of surgical resection [6,7]. To reduce postoperative recurrence and decrease the burden of micrometastases, cisplatin-based neoadjuvant chemotherapy (NAC) regimens have been used for management, but the resulting systemic toxicity reactions and potentially fatal surgical delays associated with NAC cannot be ignored [8]. Therefore, it is necessary to identify MIBC patients who may benefit from NAC while turning to other treatment strategies for non-responsive patients [9]. Unfortunately, there are still no reliable tools available to achieve this goal and avoid the risks of under- or overtreatment [10].

Accurately predicting the risk of tumor spread to regional lymph nodes in MIBC is crucial for making effective treatment decisions, such as determining the extent of PLND and the use of NAC, as the presence of LNM is associated with disease recurrence and significantly higher cancer-related mortality [11]. The possibility of positive lymph nodes is typically assessed through preoperative imaging, with contrast-enhanced CT being the mainstay of preoperative lymph node staging. However, a large proportion of patients may have lymph node involvement that is not apparent on imaging, with a sensitivity of only 33–46% [12,13]. Traditional histopathological evaluation [14] and biomarkers [15,16] are currently suboptimal in predicting LNM status, limited by high intra- and inter-observer variability, and have yet to be applied in clinical practice. Thus, there is an urgent need to find a new approach that can accurately and objectively determine the LNM status to optimize treatment decisions for MIBC patients.

Deep learning, a technique in the field of artificial intelligence (AI) that can identify subtle patterns in complex pathological images, has broad applications in computational pathology [17]. In bladder cancer, AI-based methods can be used to detect histological patterns such as malignant tumor potential [18], tumor stroma ratio [19], and tumor-infiltrating lymphocytes [20], and have been used to predict molecular subtypes [21] and overall survival [22,23]. In breast cancer [24], colorectal cancer [25,26,27], and prostate cancer [28], deep learning has successfully predicted the LNM status from primary tumor specimens. To our knowledge, only one study [29] has shown the potential of deep learning to predict LNM status from digital hematoxylin and eosin (H&E)-stained slides in primary bladder cancer. However, this study used only a single public dataset, which is susceptible to bias and may not be applicable to clinical practice. In addition to being a potentially clinically useful tool, interpretable deep learning should also provide quantitative evidence for the association of histopathological features. For example, in previous studies, deep learning has been used to identify tumor heterogeneity and vascular infiltration as negative prognostic features for MIBC [22]. Furthermore, the time-consuming and labor-intensive high-quality pixel-level image annotation is also a major challenge for current AI, which seriously hinders the development of AI in pathology [30].

Hence, in this study, we developed a weakly-supervised deep learning model to predict LNM status from routine H&E-stained slides of primary MIBC and attempted to identify new histopathological features. We validated the effectiveness and robustness of the deep learning model in three independent cohorts and compared it with the logistic regression model constructed from clinical data. Finally, we evaluated the combined classifier consisting of deep learning and clinical data.

## 2. Materials and Methods

### 2.1. Patient Cohorts

**Figure 1:**
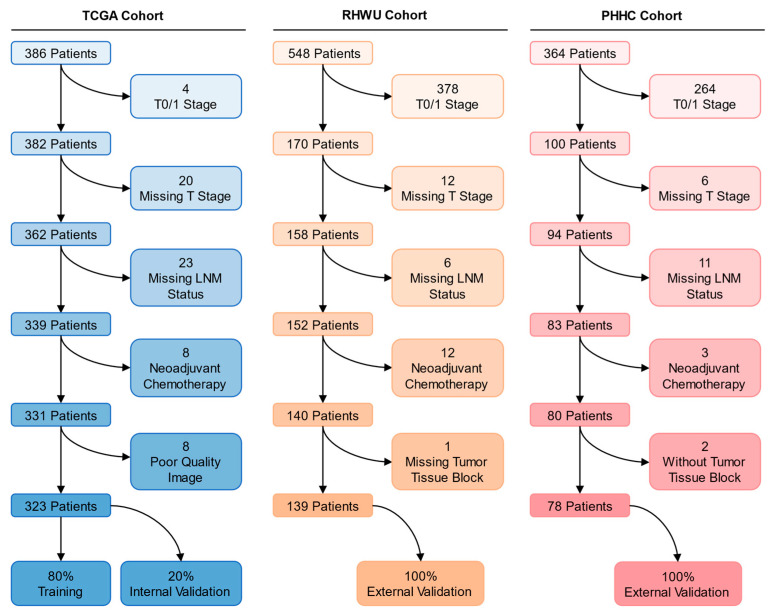
Description of pathways to recruit patients from the TCGA, RHWU, and PHHC cohorts. LNM, lymph node metastasis; TCGA, The Cancer Genome Atlas; RHWU, Renmin Hospital of Wuhan University; PHHC, People’s Hospital of Hanchuan City.

In this multicenter study, we retrospectively collected three independent cohorts to improve the generalization of the model for achieving clinical utility. We utilized the publicly available cohort of patients from The Cancer Genome Atlas (TCGA), a large international multicenter study that included patients with stage I–IV bladder cancer from 1999 to 2013 and consisted of 457 whole slide images (WSIs) from 386 cases. In the Renmin Hospital of Wuhan University (RHWU; Wuhan, China) cohort, we continuously collected 548 candidate patients from 2017 to 2023 with corresponding formalin-fixed and paraffin-embedded tumor tissue blocks (resected by surgery). Additionally, tumor tissue blocks and clinical data from 364 candidate patients were collected from 2014 and 2022 at People’s Hospital of Hanchuan City (PHHC; Hanchuan, China). Only cases with a definitive pathological diagnosis of MIBC, available pathological images or blocks, known LNM status, and clinical data regarding age, gender, T stage, lymphovascular invasion, histologic grade, and without NAC were included in all cohorts. The patient recruitment pathway is shown in Figure 1.

### 2.2. Image Preprocessing

For the RHWU and PHHC cohorts, we obtained formalin-fixed and paraffin-embedded tumor tissue blocks from each patient and then prepared H&E-stained slides. Subsequently, all slides were scanned into WSIs at 20× magnification (0.5 µm per pixel) using a digital scanner (KF-PRO-020, KFBIO Co., Ltd., Ningbo, China) and carefully reviewed by a pathologist. In principle, each patient in the RHWU and PHHC cohorts had 1 to 4 representative WSIs, while each patient in the TCGA cohort had 1 to 9 representative WSIs. The labels for multiple WSIs of the same patient were consistent.

Considering that the WSIs from the three cohorts have different magnifications, we uniformly loaded the images at 20× magnification and automatically detected the foreground using the Otsu-based method (maximal variance between-class) to segment the tissue regions. After segmentation, we used the openslide-python toolkit (https://openslide.org/, accessed on 7 January 2023) to crop patches with a size of 448 × 448 pixels for each WSI from within the segmented foreground while recording the coordinates of each patch and the corresponding WSI-level information. A color thresholding method was employed to exclude potentially mixed blank images (background pixels exceeding 80%) from all patches. Since the WSIs from the three cohorts exhibited staining differences, we used the structure-preserving color normalization method proposed by Vahadane [31] and Anand [32] to reduce image heterogeneity.

### 2.3. Feature Extraction and Reduction

Feature extraction is an important component of recognition tasks and can be used to predict performance and reduce computational requirements by utilizing feature selection. We extracted 2048 relevant features for each patch using a ResNet-50 neural network [33] with ImageNet pre-trained weights. With the emergence of novel feature selection methods, it is possible to alleviate the impact of the curse of dimensionality and facilitate the understanding of data [34,35]. To avoid the potential overfitting, long computation time, and heavy memory usage, we used an adaptive encoder for dimensionality reduction, reducing the 2048 dimensions extracted from ResNet-50 to 512 dimensions. This adaptive encoder consisted of a hidden layer architecture with 512 neurons, trained on a total of 64,600 patches (200 patches randomly selected from each WSI) over 100 epochs. After 100 epochs, the mean-squared error loss converged to 0.003.

### 2.4. Slide-Based Lymph Node Predictor (SBLNP)

SBLNP is an end-to-end weakly-supervised deep learning model, an advanced binary classification network based on multiple instance learning (MIL) and attention mechanism derived from our previously developed diagnostic and prognostic models [22].

Each WSI from three cohorts can be regarded as a bag composed of a large number of instances, which are patches segmented from the WSI. For a positive bag, there must be at least one instance classified as positive by MIL. In contrast, for the negative bag, all instances must be classified as negative. Given a bag, each instance is assigned a probability of being positive and ranked accordingly. If the bag is positive, the instances with a high probability of being positive should be close to 1, whereas if the bag is negative, all instances should be close to 0. 

**Figure 2:**
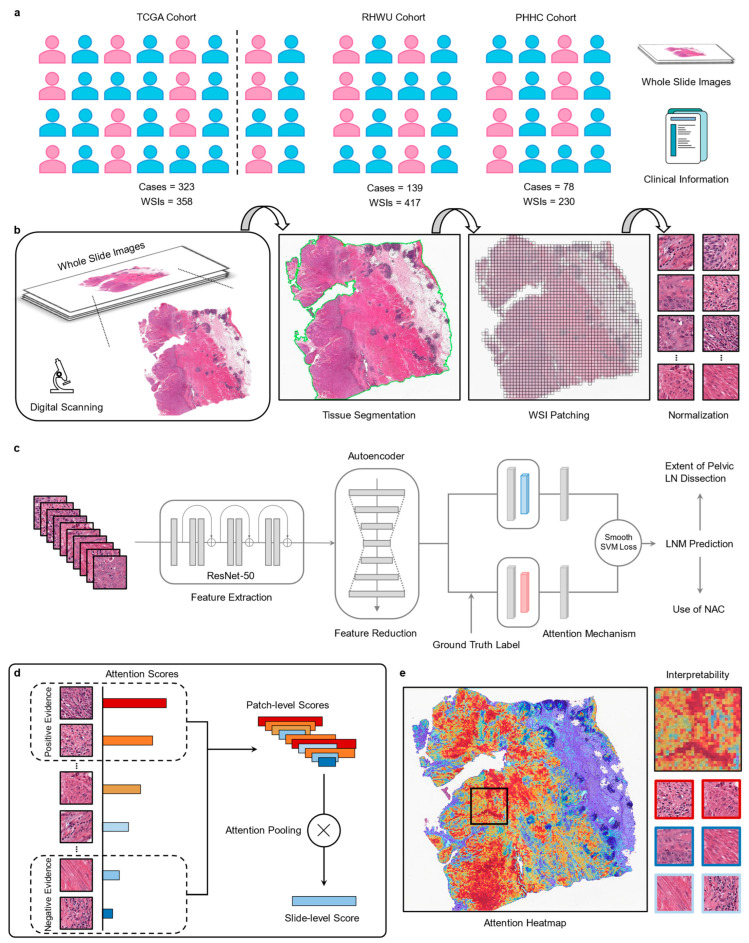
Data and workflow of the deep learning-based model. (**a**) We collected WSIs and clinical information from three cohorts of MIBC patients for the development of a predictive model. (**b**) All WSIs were digitized, then segmented into fixed-size patches, and staining differences were normalized. (**c**) We used the Resnet-50 model to extract the features of all patches and input them into MIL with an attention mechanism for training and validation after dimensionality reduction. (**d**) The attention mechanism assigned scores to each patch and ranked them, and then weighed them through the attention pooling to get the WSI-level scores. (**e**) All patches were visualized as a heatmap to identify the important histological features used for prediction. Red represented strong contributions, while blue represented weak contributions. WSIs, Whole Slide Images.

Unlike previous average- or max-pooling aggregation functions, SBLNP uses an attention-based pooling function [36] to construct a WSI-level representation by aggregating patch-level attention scores assigned during training and inference while providing interpretability. We adopted the binary smooth top-1 SVM loss [37] as the loss function for SBLNP, which proved to be more robust to noise and overfitting issues than the widely used cross-entropy classification loss. To train SBLNP, we used a five-fold cross-validation strategy for repeated validation to prevent overfitting. The Adam optimizer with an initial learning rate of 1 × 10^−4^ and ℓ2 weight decay of 1 × 10^−5^ was used to update the training weights and parameters of SBLNP. The remaining hyperparameters were set to β1 of 0.9 and β2 of 0.999. We set the maximum epoch during training to 200, and when the loss of SBLNP did not change for 20 consecutive epochs, we used early stopping to stop training and saved the best model for internal and external validation. A simplified layout of the SBLNP is shown in Figure 2.

### 2.5. Interpreting Predictions via Attention Heatmap

To explain the degree of attention of the SBLNP in different regions of WSI, we saved the attention scores of all patches. These scores represented their importance to the entire WSI and were converted to percentages, normalized, and scaled between 0 and 1. Scores closer to 1 indicated a greater contribution to prediction, while scores closer to 0 indicated a smaller contribution. We then used diverging colormap to convert the normalized scores into RGB colors and mapped them to the original WSI based on the saved spatial position information to generate an attention heatmap, with a transparency value set to 0.6 to facilitate simultaneous visualization of the underlying histological features. Red represented the areas with high attention by the SBLNP (positive evidence), while blue represented low attention (negative evidence).

### 2.6. Quantification of Histopathological Features

In addition to gaining deeper insights into the histopathological features identified by SBLNP as predictive of LNM status through heatmap visualization, we performed a blinded observer study with a pathologist. We randomly extracted 100 WSIs (LNM-positive:LNM-negative = 1:1) and then selected the 15 top-scoring patches from each WSI and aggregated them together. A total of 1500 patches were obtained from LNM-positive and -negative cases and were reviewed by a pathologist in a blinded manner, with recorded histological features for quantitative comparison.

### 2.7. Clinical Classifier and Combined Classifier (Clinical Classifier + SBLNP)

For the clinical classifier, we performed logistic regression analyses based on clinical data of patients regarding age, gender, T stage, Lymphovascular invasion (LVI), and histologic grade, which were available in all three cohorts. In addition, we constructed a combined classifier encompassing the above variables and integrating SBLNP scores. The T stage used in this study was the pathological T stage (pT stage) rather than the clinical T stage. The datasets used for both classifiers were consistent with the training and internal/external validation datasets of SBLNP for analysis and comparison, and a five-fold cross-validation strategy was also carried out.

### 2.8. Statistical Analysis

The performance of three classifiers (Clinical classifier, SBLNP and SBLNP + Clinical classifier) was evaluated by calculating the area under the receiver operating characteristic (AUROC) on internal and external validation sets. The 95% confidence interval (CI) of the internal validation set was obtained by five-fold cross-validation, while the 95% CIs of the external validation sets were calculated from five repeated experiments. Statistical significance was assessed using DeLong’s test [38] among the three classifiers. Categorical variables were described as number (percentage), and continuous values were described as the median (extremum). A two-sided *p*-value less than 0.05 was considered statistically significant.

## 3. Results

### 3.1. Patient Characteristics

After the screening, we included 358 WSIs from 323 MIBC patients in the TCGA cohort, 417 WSIs from 139 MIBC patients in the RHWU cohort, and 230 WSIs from 78 MIBC patients in the PHHC cohort. Among these, 80% of the WSIs (*n* = 286) from the TCGA cohort were used as a training set for model development, and the remaining 20% (*n* = 72) were used as an internal validation set. The RHWU and PHHC cohorts were used as external validation sets to test the generalization performance of the SBLNP. The detailed data distribution is shown in Supplementary Tables S1 and S2. Table 1 displays the characteristics of the included patients in the three cohorts.

### 3.2. Performance of the SBLNP

**Figure 3:**
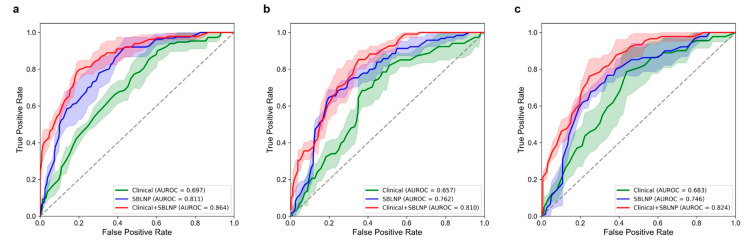
Performance of the clinical classifier, the SBLNP, and the combined classifier in (**a**) the TCGA cohort, (**b**) the RHWU cohort, and (**c**) the PHHC cohort. SBLNP, Slide-Based Lymph Node Predictor.

The SBLNP achieved an AUROC of 0.811 (95% CI, 0.771–0.855; Table 2, Figure 3) on the internal validation set (TCGA cohort). On the external validation sets (RHWU cohort and PHHC cohort), its predictive performance was robust as it achieved decent AUROCs of 0.762 (95% CI, 0.725–0.801) and 0.746 (95% CI, 0.687–0.799), respectively.

### 3.3. Performance of the Clinical Classifier

The clinical classifier purely based on patient information yielded an AUROC of 0.811 (95% CI, 0.661–0.728; Table 2, Figure 3) on the internal validation set (TCGA cohort). In the RHWU cohort and the PHHC cohort, the AUROCs were 0.657 (95% CI, 0.595–0.713) and 0.683 (95% CI, 0.537–0.829), respectively, and their performance was lower than that of the SBLNP.

### 3.4. Performance of the Combined Classifier (Clinical Classifier + SBLNP)

We achieved the best predictive performance when we incorporated the prediction scores from SBLNP into the logistic regression model. We calculated coefficients and odds ratios (ORs) for each input variable in the logistic regression model to roughly quantify the strength of the association with LNM status (Table 3). The results showed that LVI (ORs = 1.379; *p* < 0.001), pT stage (ORs = 1.113; *p* = 0.005), and SBLNP-based score (ORs = 2.072; *p* < 0.001) were significantly associated with LNM status, among which the SBLNP achieved highest coefficient. The combined classifier yielded an AUROC of 0.864 (95% CI, 0.827–0.906; Table 2, Figure 3) on the internal validation set (TCGA cohort). In the RHWU cohort and the PHHC cohort, the AUROCs of the combined classifier were 0.810 (95% CI, 0.780–0.844) and 0.824 (95% CI, 0.788–0.861), which outperformed the clinical classifier significantly (Delong’s test, *p* = 0.004 and *p* = 0.021, respectively; Table 4). Together, the SBLNP is a strong predictor of LNM status in addition to established clinicopathological features.

### 3.5. Visualizing Deep Learning-Based Predictions

**Figure 4:**
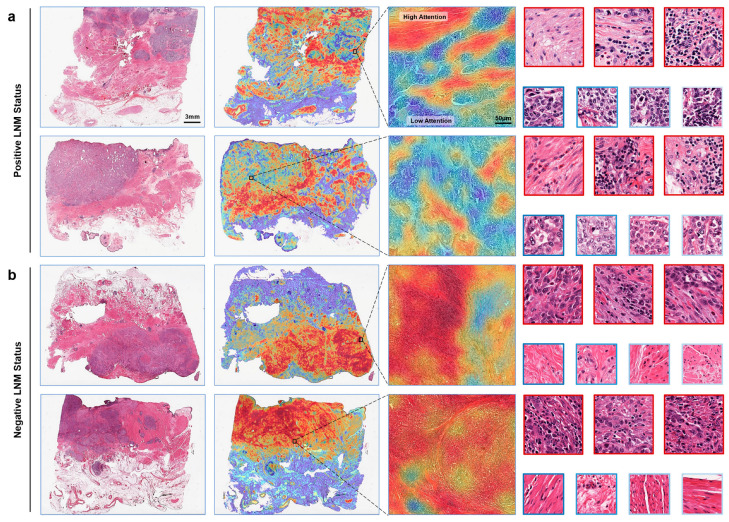
Heatmaps of (**a**) LNM-positive and (**b**) LNM-negative patients from the TCGA cohort predicted correctly by the SBLNP. The attention scores of all patches are calculated by the SBLNP, and an attention heatmap corresponding to each WSI is generated and overlaid on it. Then the local area is further zoomed in to highlight the high- (red) and low-attention (blue) histopathological features. LNM, lymph node metastasis.

**Figure 5:**
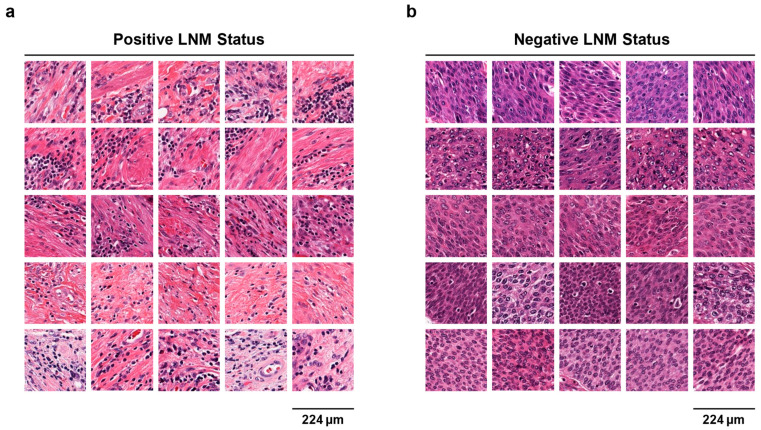
The 5 top-scoring patches of (**a**) the 5 top-scoring LNM-positive and (**b**) 5 top-scoring LNM-negative patients in the TCGA cohort. LNM, lymph node metastasis.

Next, we intended to use visualization techniques to understand how the deep learning-based model predicted, thereby guiding pathologists to discover potentially relevant recognition patterns. To achieve this goal, the SBLNP with an attention mechanism assigned each patch an attention score, which is used to represent its contribution to the prediction. We then converted the scores into a heatmap to intuitively understand the region of interest of the model. This visualization method allowed us to identify the pathological patterns most associated with LNM-positive or -negative status. As shown in Figure 4, for LNM-positive WSIs, the model visually paid more attention to the stromal region around the tumor; on the contrary, for LNM-negative WSIs, the model focused more on bladder cancer itself. In addition, Figure 5 presented the five top-scoring patches for each of the five LNM-positive and five LNM-negative WSIs in the TCGA cohort. Regarding LNM-positive patches, we observed that the tissue regions yielding high prediction scores were predominantly located in the surrounding non-neoplastic tissue rather than the bladder cancer itself. Some patches contained inflammatory cell infiltration, mainly composed of lymphocytes. Conversely, tumor cells were dominant in patches with high LNM-negative prediction.

### 3.6. Quantitative Assessment of Histopathological Features

To further confirm the above findings, we performed a quantitative study of 100 WSIs from LNM-positive and -negative patients. The 15 top-scoring patches were obtained from each WSI, for a total of 1500 patches, and were reviewed by a pathologist blinded to the LNM status in advance. We found that ‘immune cells’ and ‘stroma’ were the most abundant tissue classes in LNM-positive patients. Conversely, ‘tumor cells’ was the most abundant tissue class in LNM-negative patients (Table 5). Surprisingly, most of the LNM-positive patches were predominantly located in the stromal region rather than the tumor region (ratio LNM-positive patches in stroma = 336/750), which was in complete contrast to the LNM-negative results (ratio LNM-negative patches in stroma = 16/750, Chi-squared test, *p* < 0.001). In the LNM-negative group, tumor tissue-related patches accounted for the largest proportion (ratio LNM-negative patches in tumor = 654/750), which also produced the contrary result to the LNM-positive group (ratio LNM-positive patches in tumor = 45/750, Chi-squared test, *p* < 0.001). More precisely, features of the tumor tissue itself do not seem to be too important in the process of predicting LNM-positive status, while the microenvironment around the tumor may need more attention. Finally, we performed a more fine-grained analysis of the “stroma” patches and found that in the LNM-positive group, stroma with lymphocytic inflammation (241/336) outnumbered those without inflammation (95/336) by more than 150%. Conversely, in the LNM-negative group, only 16/750 patches belonged to the stroma, and these patches did not show any inflammatory infiltration.

## 4. Discussion

The presence of LNM in MIBC patients has been shown to be associated with a poorer prognosis, and the 5-year overall survival of LNM-positive patients is significantly lower than that of LNM-negative patients [39,40,41]. Therefore, predicting the LNM status before RC is crucial for clinical decision-making in MIBC, particularly regarding the extent of PLND and the use of NAC [42,43]. However, the existing tools for predicting LNM status are not satisfactory in terms of accuracy and reliability, with some patients being under or over-staged [13,44,45]. Hence, there is an urgent need to find more tools to accurately predict the LNM status before RC. In this study, we developed a weakly-supervised deep learning model (SBLNP) that can predict the LNM status from digital H&E-stained images of the primary MIBC. Our results demonstrated that SBLNP performed well with AUROC values between 0.7 and 0.8 in three independent cohorts, showing excellent generalization ability. In addition, the results of the clinical classifier confirmed that the LVI and pT stage were strongly associated with the LNM status [46,47], and the combined classifier based on SBLNP and clinicopathologic variables demonstrated satisfactory performance.

Previous studies have shown that cisplatin-based NAC can serve as first-line adjuvant therapy for MIBC and prolong survival, with an absolute increase of 8% in the 5-year survival [48] and from 30% to 36% in the 10-year survival [49]. In addition, the European Association of Urology guidelines recommend NAC for MIBC patients with T2-T4a and cN0M0 before RC [10]. However, NAC has not been widely used in clinical practice, with only 19% of patients receiving NAC before RC [50], mainly due to a lack of response to treatment, the potential for surgical delays, and the risks of over-staging and over-treatment. Moreover, MIBC patients with LNM-positive may benefit from NAC, as the goal of NAC is to target distant metastases (including LNM) rather than control the local region [49,51].

Currently, RC and bilateral PLND are the standard treatment for MIBC patients, but the optimal extent of PLND remains controversial. Previous studies have shown that the extent of LNM in some patients exceeds the region of the standard PLND template [52,53], and a larger dissection area is needed to achieve better regional control and more accurate lymph node staging [4]. Another systematic review indicated that extended PLND may provide more therapeutic benefits than standard PLND [54]. However, the use of extended PLND in clinical practice is limited by the increased surgical difficulty, potential surgical risks, and complications.

In clinical practice, the identification of MIBC patients with a high risk of LNM before RC will aid in determining an appropriate population for NAC and extended PLND. Thus, there is an urgent need for the development of new tools for accurate prediction of LNM status before RC to optimize clinical treatment decisions.

The use of radiomics and genomics to predict the LNM status [55,56,57] has been shown to achieve good performance. However, the cohort used by these methods is single, the amount of data is small, and the generalization performance of the model still needs to be verified. Despite similar studies based on deep learning in breast cancer [24], colorectal cancer [25,26,27], and prostate cancer [28], the small training data size, poor interpretability, and time-consuming and labor-intensive manual annotation remain obstacles to their clinical application. Therefore, developing a weakly-supervised or unsupervised model with high interpretability and generalization ability is the starting point of this study. In this study, the integration of clinicopathologic information and SBLNP contributes to the improvement of predictive model performance, indicating that they are not redundant with each other. Our SBLNP model requires only H&E-stained pathology slides as input to obtain predictions related to LNM status, which is cost-effective since such slides are readily available in the surgical setting. Interestingly, based on the interpretability of our model, we found that the stromal region around the tumor may contain histological information related to the LNM status. This also reflects that previous studies may have lost important predictive information by only training the model on the annotated tumor region. Through quantitative analysis and blind evaluation of the 1500 top-scoring patches, we validated the above observations. Specifically, a key predictive feature appears to be the stroma with lymphocytic inflammation around the tumor.

To our knowledge, this is the first study that links inflammatory infiltrate in the stroma of MIBC to the LNM status. Improved tumor microenvironment assessment and intervention are critical to the development of effective therapies for bladder cancer [58]. Further mechanistic studies are needed to validate the predictive patterns of deep learning. Previous studies have shown that tumor-associated inflammation in the tumor microenvironment may be related to the tumor’s biological behavior and prognosis and may serve as a potential predictor for LNM [59,60]. Lymphocytes and monocyte-differentiated tumor-associated macrophages are involved in the composition of the tumor microenvironment, and their interaction can promote tumor angiogenesis and invasion [61]. In addition, a large number of cancer-associated fibroblasts (CAFs) in the stroma affect the occurrence of LNM. Recently, in cervical squamous cell carcinoma, CAFs were observed to promote LNM through the destruction of the lymphatic endothelial barrier in cell-level experiments [62]. In gastric cancer, the relationship between interleukin-8, CAFs, and lymphocytes was revealed through cell and animal experiments, explaining the possible mechanism of LNM occurrence [63]. Therefore, further elucidation of these mechanisms may improve our understanding of the ability of deep learning to predict LNM in MIBC.

Despite the advantages of our study, limitations exist. First, our study is retrospective and requires larger multicenter prospective studies to further confirm its clinical value. Second, the variables included in our clinical classifier are not comprehensive enough, and clinical information, including tumor size, location, and tumor budding, needs to be collected. Our combined classifier achieved the best performance; integrating pathological features with clinical key information is another important direction. In addition, the inclusion of LNM-positive and -negative categories in this study is unbalanced, as data with better class balance can help improve performance. Finally, we found that a few artifacts and out-of-focus tissues are still mixed in the training data, and future research needs to further control the quality of the data to exclude possible interfering factors.

## 5. Conclusions

We developed a weakly-supervised deep learning model for predicting LNM status in MIBC patients. In three independent cohorts, the SBLNP demonstrated decent predictive performance. Interestingly, the SBLNP generated a new biological hypothesis, defining the lymphocytic inflammatory stroma as a key factor for prediction, but further experimental validation is needed. Future efforts are needed to improve SBLNP for early clinical implementation, providing precise guidance for the use of NAC and the extent of PLND in MIBC patients.

## Figures and Tables

**Table 1:** Clinical, biological, and pathological characteristics of MIBC patients included in the TCGA, RHWU, and PHHC cohorts.

	TCGA (*n* = 323)	RHWU (*n* = 139)	PHHC (*n* = 78)
Age (years)	69 (34, 90)	66 (26, 87)	70 (45, 90)
Gender			
Female	88 (27.24%)	21 (15.11%)	17 (21.79%)
Male	235 (72.76%)	118 (84.89%)	61 (78.21%)
pT stage			
pT2	98 (30.34%)	67 (48.20%)	30 (38.46%)
pT3	175 (54.18%)	44 (31.65%)	28 (35.90%)
pT4	50 (15.48%)	28 (20.14%)	20 (25.64%)
pM stage			
pM0	146 (45.20%)	104 (74.82%)	57 (73.08%)
pM1	7 (2.17%)	35 (35.18%)	21 (26.92%)
pMx	170 (52.63%)	0 (0%)	0 (0%)
pTNM stage			
Stage II	83 (25.70%)	58 (41.73%)	74 (32.18%)
Stage III	122 (37.77%)	45 (32.37%)	78 (33.91%)
Stage IV	118 (36.53%)	36 (25.90%)	78 (33.91%)
Histologic grade			
High grade	303 (93.81%)	129 (92.81%)	74 (94.87%)
Low grade	18 (5.57%)	10 (7.19%)	4 (5.13%)
Missing	2 (0.62%)	0 (0%)	0 (0%)
LVI			
No	104 (32.20%)	81 (58.27%)	47 (60.26%)
Yes	127 (39.32%)	58 (41.73%)	31 (39.74%)
Missing	92 (28.48%)	0 (0%)	0 (0%)
LN status			
Negative (pN0)	207 (64.09%)	102 (73.38%)	53 (67.95%)
Positive (pN1-3)	116 (35.91%)	37 (26.62%)	25 (32.05%)
LNs examined number	18 (1, 170)	21 (1, 64)	16 (1, 47)
Positive LNs number	2 (1, 97)	2 (1, 20)	3 (1, 31)
Survival status			
Alive	178 (55.11%)	-	-
Dead	145 (44.89%)	-	-
OS time (months)	17.4 (0, 165.6)	-	-

MIBC, Muscle-invasive Bladder Cancer; TCGA, The Cancer Genome Atlas; RHWU, Renmin Hospital of Wuhan University; PHHC, People’s Hospital of Hanchuan City; LVI, Lymphovascular Invasion; LN, Lymph Node; OS, overall survival.

**Table 2:** The AUROCs of clinical classifier, SBLNP, and combined classifier (Clinical + SBLNP) in internal and external validation sets.

Model	TCGA Cohort	RHWU Cohort	PHHC Cohort
	AUROC (95% CI)	AUROC (95% CI)	AUROC (95% CI)
Clinical	0.697 (0.661, 0.728)	0.657 (0.595, 0.713)	0.683 (0.537, 0.829)
SBLNP	0.811 (0.771, 0.855)	0.762 (0.725, 0.801)	0.746 (0.687, 0.799)
Clinical + SBLNP	0.864 (0.827, 0.906)	0.810 (0.780, 0.844)	0.824 (0.788, 0.861)

CI, Confidence Interval; TCGA, The Cancer Genome Atlas; RHWU, Renmin Hospital of Wuhan University; PHHC, People’s Hospital of Hanchuan City; SBLNP, Slide-Based Lymph Node Predictor.

**Table 3:** Coefficients, *p* values, and odds ratios with 95% CIs for all variables of the logistic regression model of the combined classifier (Clinical + SBLNP) in the TCGA cohort.

Characteristic	Coefficient	*p* Value	Odds Ratio (95% CI)
Age	0.0034	0.120	1.003 (0.999–1.008)
Gender	0.0591	0.256	1.061 (0.958–1.175)
LVI	0.3211	<0.001	1.379 (1.252–1.518)
pT stage	0.1072	0.005	1.113 (1.033–1.199)
Histologic grade	−0.0808	0.474	0.922 (0.739–1.151)
SBLNP	0.7285	<0.001	2.072 (1.694–2.535)

CI, Confidence Interval; TCGA, The Cancer Genome Atlas; LVI, Lymphovascular Invasion; SBLNP, Slide-Based Lymph Node Predictor.

**Table 4:** Performance comparisons (DeLong’s test) between clinical classifier, SBLNP, and combined classifier (Clinical + SBLNP) in internal and external validation sets.

Comparisons	TCGA Cohort	RHWU Cohort	PHHC Cohort
Clinical vs. SBLNP	*p* = 0.028	*p* = 0.632	*p* = 0.703
Clinical vs. Clinical + SBLNP	*p* = 0.001	*p* = 0.004	*p* = 0.021
SBLNP vs. Clinical + SBLNP	*p* = 0.093	*p* = 0.014	*p* = 0.005

TCGA, The Cancer Genome Atlas; RHWU, Renmin Hospital of Wuhan University; PHHC, People’s Hospital of Hanchuan City; SBLNP, Slide-Based Lymph Node Predictor.

**Table 5:** Histopathological quantitative analysis of the 1500 top-scoring patches.

	Positive LNM Status		Negative LNM Status	
Histological Features	*n* Patches	% Patches	*n* Patches	% Patches
Tumor cells	45	6	654	87.2
Normal transitional epithelium	23	3.07	27	3.6
Muscle tissue	82	10.93	24	3.2
Adipose tissue	8	1.07	1	0.13
Immune cells	218	29.06	3	0.4
Necrotic tissue	29	3.87	18	2.4
Stroma	336	44.8	16	2.13
Out of focus	9	1.2	7	0.94

## Data Availability

The datasets of the TCGA cohort for this study can be found in the [The Cancer Genome Atlas Program] [https://portal.gdc.cancer.gov/, accessed on 7 January 2023].

## Supplementary Materials

The following supporting information can be downloaded at: https://www.mdpi.com/article/10.3390/cancers15113000/s1, Table S1: Dataset distribution of patients and corresponding images in the predictor (SBLNP). Table S2: Dataset distribution of images in the training, internal validation, and external validation sets.
