# Supplementary material for: Predicting Lymph Node Metastasis Status from Primary Muscle-Invasive Bladder Cancer Histology Slides Using Deep Learning: A Retrospective Multicenter Study
Source: Cancers (Basel). 2023 May 31;15(11):3000. doi: 10.3390/cancers15113000 (PMC10251851; doi:10.3390/cancers15113000)
Supplement: Supplementary file 1 [file cancers-15-03000-s001.zip › cancers-2379823-supplementary.pdf]

Supplementary Materials: Predicting Lymph Node Metastasis Status from Primary Muscle-Invasive Bladder Cancer Histology Slides Using Deep Learning: A Retrospective Multicenter Study

Qingyuan Zheng, Jun Jian, Jingsong Wang, Kai Wang, Junjie Fan, Huazhen Xu, Xinmiao Ni, Song Yang, Jingping Yuan, Jiejun Wu, Panpan Jiao, Rui Yang, Zhiyuan Chen, Xiuheng Liu and Lei Wang

Table S1. Dataset distribution of patients and corresponding images in the predictor (SBLNP).

	**Patients**	**Total Images**	**Positive LNM Images**	**Negative LNM Images**
TCGA Cohort	323	358	116	207
RHWU Cohort	139	417	111	306
PHHC Cohort	78	230	79	151

Table S2. Dataset distribution of images in the training, internal validation, and external validation sets.

	**TCGA Cohort**		**RHWU Cohort**	**PHHC Cohort**
	Training set	Internal validation set	External validation set	External validation set
SBLNP	286	72	417	230
